# Role of PI3K/Akt/mTOR pathway in mediating endocrine resistance: concept to clinic

**DOI:** 10.37349/etat.2022.00078

**Published:** 2022-04-24

**Authors:** Aglaia Skolariki, Jamie D’Costa, Martin Little, Simon Lord

**Affiliations:** 1 Department of Oncology, University of Oxford, Churchill Hospital, OX3 7LE Oxford, UK; 2 Department of Oncology, Churchill Hospital, OX3 7LE Oxford, UK; University of Aberdeen, UK

**Keywords:** Breast cancer, endocrine therapy, PI3K/Akt/mTOR pathway

## Abstract

The majority of breast cancers express the estrogen receptor (ER) and for this group of patients, endocrine therapy is the cornerstone of systemic treatment. However, drug resistance is common and a focus for breast cancer preclinical and clinical research. Over the past 2 decades, the PI3K/Akt/mTOR axis has emerged as an important driver of treatment failure, and inhibitors of mTOR and PI3K are now licensed for the treatment of women with advanced ER-positive breast cancer who have relapsed on first-line hormonal therapy. This review presents the preclinical and clinical data that led to this new treatment paradigm and discusses future directions.

## Introduction

Endocrine therapy (ET) remains a key systemic treatment for both early and advanced breast cancer. Over the past 40 years, 3 main classes of ET have demonstrated clinical benefit and been licensed by the Food and Drug Administration (FDA) for the treatment of breast cancer; selective estrogen receptor modulators (SERMs; tamoxifen and toremifene); selective estrogen receptor degraders (SERDs; fulvestrant); and aromatase inhibitors (AI, letrozole, anastrozole, and exemestane) [1, 2]. However, resistance to ET in estrogen receptor-positive (ER^+^) breast cancer is common and remains a significant clinical challenge. A number of endocrine resistance mechanisms have been evaluated in the laboratory and clinic but to date only targeting the cyclin-dependent kinases 4 and 6 (CDK4/6) and PI3K/Akt/mTOR signaling axes has successfully translated to licensed drugs. In this review, we describe the multiple agents that target the PI3K/Akt/mTOR pathway which has and is being evaluated in the clinic for the treatment of breast cancer but with variable success. In particular, the selection of patients and drug toxicity have both proved to be challenged in the clinical development of this treatment class.

## Discovery of the PI3K/Akt/mTOR pathway

The PI3K/Akt/mTOR pathway is a key intracellular signal transduction pathway that has provoked great interest as a therapeutic target in cancer [3]. This signaling cascade is implicated in tumorigenesis via activation of downstream signaling that regulates cellular proliferation, survival, metabolism, angiogenesis, and increasing motility [4]. In 1988 the Cantley group identified the agent that catalyzed the phosphorylation of phosphatidylinositol 4,5-bisphosphate (PIP_2_) into phosphatidylinositol-3,4,5-trisphosphate (PIP_3_), naming this enzyme PI3K [5], and subsequently, insulin signaling was found to regulate PI3K activity [6]. The design of oligonucleotide probes enabled the isolation of the first complementary DNA (cDNA) of a PI3K catalytic subunit, named p110α [7]. The discovery that activation of the PI3K pathway could be promoted by rat sarcoma (RAS) and insulin signaling, offered insight into how the PI3K pathway was involved in cell growth and proliferation [3, 8–10]. The serendipitous discovery that wortmannin, a mold metabolite inhibited PI3K signaling and the creation of a first synthetic inhibitor of PI3K, led to further research into the role that PI3K has in regulating cellular metabolism, and in particular glucose uptake and chemotaxis [3, 11–13]. Subsequently, the PI3K pathway was implicated in tumorigenesis with somatic mutations in *PIK3CA* genes found in several tumor types [14].

## PI3K/Akt/mTOR signaling

**Figure 1.**
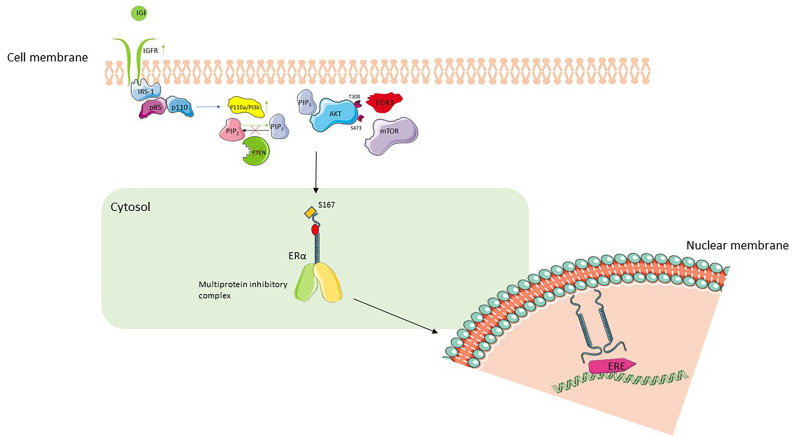
PI3K/Akt/mTOR pathway and interaction with ER signaling. The binding of IGF to IGFR leads to autophosphorylation of the receptor and IRS-1. IRS-1 leads to splitting of heterodimeric complex (p85, p110) and activation of p110 which via its PI3K activity converts PIP_2_ to PIP_3_. PIP_3_ translocates to the plasma membrane and binds to Akt, allowing phosphorylation at T308 by PDK1 which achieves partial activation of Akt. Full activation of Akt is achieved by phosphorylation at S473 by mTOR. Active Akt phosphorylates S167 facilitate activation of downstream ER nuclear transcriptional activity [39]. Upregulation of IGFR, constitutively active PI3K or loss of PTEN leads to activation of PI3K/Akt/mTOR pathway, increased ligand-independent activation of ER, and resistance to ET. IGF: insulin growth factor; IGFR: IGF receptor; IRS-1: IR substrate-1; ERE: estrogen response element; PTEN: phosphatase and tensin homolog; PDK1: 3-phosphoinositide-dependent protein kinase-1; ER: estrogen receptor

In nature, there are three different classes of PI3K, yet only class I PI3K isoforms are able to produce PIP_3_ from PIP_2_, which subsequently works as a secondary cellular messenger [5, 15]. The PI3K pathway can be initiated by binding of ligands, either to transmembrane tyrosine kinase linked receptors [insulin receptor (IR) and Erb-B2 receptor tyrosine kinase 3 (ERBB3) receptor human epidermal growth factor receptor 3 (HER3)] or to G-protein-coupled receptors and subsequent RAS GTPases (Figure 1) [6, 16–19]. The heterodimeric complex of p85–p110 determines PI3K activity, with p85 having no intrinsic PI3K activity and thus acting to stabilize the complex and inhibit PI3K activity [20–23]. Upon binding of the ligand to the receptor, cellular phosphoproteins bind to the Src homology 2 (SH2) domain of p85, inducing a conformational change and leading to the release of p85 from p110 [23–25]. Activated p85 (p85a) can have downstream effects via the mitogen-activated protein kinase (MAPK)-extracellular signal-regulated kinase (ERK) pathways to cause cellular proliferation and increase cellular motility [26]. ERα is methylated by the arginine methyltransferase protein arginine methyltransferase 1 (PRMT1) and this has been shown to be a prerequisite for the formation of the methylated ERα/Src/PI3K complex and for the activation of downstream signaling [27].

p110a/PI3K catalyzes the phosphorylation of PIP_2_ into PIP_3_ [5] whilst the reverse of this reaction, the dephosphorylation of PIP_3_ to PIP_2_, is catalyzed via PTEN [28]. PIP_3_ translocates to the plasma membrane and binds to the pleckstrin homology domain (PHD) of Akt/protein kinase B (PKB) [29] and this allows partial activation of Akt by facilitating the phosphorylation of Akt on threonine 308 (T308) by PDK1 [30]. Full activation of Akt occurs upon binding of mTOR to the serine 473 (S473) carboxy-terminal hydrophobic motif of Akt [31]. Fully activated Akt regulates and enhances angiogenesis, anabolic metabolism, proliferation, and inhibition of apoptosis [32].

The mTOR comprises 2 structurally different catalytic complexes, mTOR complex 1 (mTORC1) and mTORC2 [33]. mTOR regulates several cell processes including proliferation, autophagy, metabolism, survival, stress response, angiogenesis, and survival. mTORC1 phosphorylates T389 on P70-S6kI with full activation provided by the phosphorylation on T229 by PDK1 leading to transcription of pro-growth factors [34]. mTORC1 negatively inactivates autophagy by the actions of unc-51-like kinase 1/2 (ULK1/2) and autophagy-related 12 (ATG12) [35–37]. mTORC2 is involved in the action of Akt by phosphorylating S473, thus mTORC2 could be considered upstream of mTORC1 [38]. Constitutive upregulated mTOR activity leads to cancer cells with unregulated growth and inhibition to autophagy thus conferring them a survival advantage and as such targeting mTOR activity has been a therapeutic approach of great interest in a number of tumor types [38].

## Interaction between ER signaling and the PI3K/Akt/mTOR signaling

The identification of the ER as a therapeutic target led to a paradigm shift in the treatment of breast cancer and the development of a number of different classes of hormonal therapy, in particular, selective SERM, SERD, and AI [40].

The two ERs (ERα and ERβ) are derived from the receptor family of ligand activating transcription factors. ERα and ERβ are encoded by distinct genes; ERα is encoded by estrogen receptor 1 (*ESR1*), on 6q25.1 and ERβ is encoded by *ESR2*, on 14q22-24 [41, 42]. Circulating estrogen has differential downstream effects on signaling networks depending on the proportion of expression of the two isoforms and the ligands to which they bind. ERα is more widely expressed than ERβ and while ERβ is thought to have antiproliferative actions, it is believed ERα activation leads to proliferation in breast cancers and is the main focus of the review article. In the absence of estrogen, ERα is held in an inactive ER-chaperon complex [43]. Activation of downstream signaling by ER may be ligand-dependent or ligand-independent. Upon ligand binding by estrogens, ER dissociates from its chaperone proteins facilitating homodimerization or heterodimerization between the isoform occurs, and a conformational change results in binding of response elements to the regulatory region of ER regulated genes. When either isoform of ER is bound to DNA, protein complexes form to either activate or repress target genes [43, 44].

Ligand independent activation of the ER can be mediated by intracellular/extracellular signals such as the epidermal growth factor receptor (EGFR) and IGF-1 [45]. Here, downstream activation of the MAPK by epidermal growth factor (EGF) binding to its receptor, leads to subsequent phosphorylation of S118 on the activation function-1 (AF1) domain of ER and downstream signaling via binding of ER to ER regulatory regions on genes [46–48]. Furthermore, ligand-independent activation may occur through phosphorylation of S167 on the AF1 of ER via the actions of Akt [49].

Whole-exome sequencing by The Cancer Genome Atlas (TCGA) program has confirmed earlier work, showing that gain of function mutations in *PIK3CA* and *AKT1*, and inactivating mutations in *PTEN* are common in ER^+^ breast cancer (32–49%, 13–24%, and approximately 7% respectively) [50–52]. The common mutational hotspots of *PIK3CA* are in the kinase and helical domain which are understood to increase kinase activity thus driving cell proliferation [51, 53]. A meta-analysis of 26 studies encompassing 4,754 patients demonstrated that *PIK3CA* mutations are strongly associated with ER expression [odds ratio (OR) 1.92, 95% confidence interval (CI): 1.65–2.23; *P* < 0.00001] [54]. Overall clinical data suggest that *PIK3CA* mutations in ER^+^ tumors, may be a favorable prognostic marker [55, 56]. Murine models have demonstrated that a constitutively active Akt can lead to breast cancer tumorigenesis, and in human samples, 60% of ductal carcinoma *in situ* demonstrated Akt overexpression [57, 58]. Furthermore constitutively activated Akt was found to confer resistance to both SERM and SERD therapy by causing estrogen-independent activation of ER [59]. *PTEN* mutations causing constitutive activation of the PI3K pathway have also been implicated in tumorigenesis in murine breast cancer models and also in conferring resistance to ET [60, 61].

## Preclinical studies combining ET and inhibition of the PI3K/Akt/mTOR pathway

In the context of ER^+^ breast cancer, the rationale for targeting the PI3K/Akt/mTOR pathway is to overcome resistance to ET by reducing proliferation driven by ligand-dependent activation and inhibiting ligand-independent activation of the ER. Both pan-PI3K inhibitors target all four PI3K class I isoforms and isoform-specific PI3K inhibitors have been developed [62]. Buparlisib is a pan-class I PI3K inhibitor that demonstrated inhibition of tumor growth in a murine *PIK3CA*-mutant xenograft model [63]. Similar preclinical anti-tumor activity was observed for other class I pan PI3K inhibitors. Copanlisib (BAY 80-6946) and pictilisib (GDC-0941) induced tumor regression in a rat HER2-amplified *PIK3CA*-mutated and murine HER2 amplified *PIK3CA*-mutated breast cancer models, respectively [64, 65]. *In vitro*, fulvestrant sensitized ER^+^ breast cancer cells to PI3K inhibition and subsequently induced apoptosis [66].

The potent, allosteric Akt inhibitor, MK2206, has demonstrated *in vitro* activity against both thyroid and breast cancer cell lines with *PI3KCA* mutations [67–71] and, in a breast cancer *PTEN* mutated xenograft model, MK2206 inhibited tumor growth [68]. Capivasertib (AZD5363), an ATP-competitive Akt inhibitor has demonstrated anti-tumor activity in a xenograft model of *PIK3CA*-mutated breast cancer [72]. High Akt activity was found to be a predictive biomarker of sensitivity to ipatasertib (GDC-0068) in *PTEN*/*PIK3CA*-mutant MCF breast cancer cells [73].

In a screen of breast cancer cell lines, PTEN, Akt, and phosphorylated ribosomal S6 kinase 1 (pS6K1) levels were associated with sensitivity to the mTOR inhibitor, rapamycin [74]. Insulin and IGF signaling are implicated in breast cancer tumorigenesis [75] and IGF binding to its receptor and subsequent autophosphorylation leads to phosphorylation of IR substrate-1 (IRS-1) and activation of the PI3K/Akt/mTOR pathway [75]. Upregulation of IGF-1 receptor (IGF-1R) by cancer cells has been observed to associate with resistance to hormonal therapy [76]. Monoclonal antibodies and ligand neutralizing strategies have been developed to target IGF-1R [77]. NT157 which targets the IRS-1 for destruction via the proteasome has demonstrated a pro-apoptotic effect in PLX4032 melanoma cells and prostate cancer cells [78, 79]. MEDI-573 is a monoclonal antibody that neutralizes IGF-1 and IGF-2 with activity in IGF-1/IGF-2 driven tumors in a murine model [80]. BMS-536924, a dual IGF-1R/IR tyrosine kinase inhibitor inhibited tumor growth in tamoxifen-resistant MCF-7 cells [81]. Finally, in a resistant ER^+^ xenograft model, there was a synergistic interaction between another IGF-1R/IR inhibitor (OSI-906) and fulvestrant [82].

In summary, the ER has complex interactions with the IGF/PI3K/Akt/mTOR signaling cascade and substantial preclinical data have demonstrated the potential for synergistic co-targeting of these pathways.

## Targeting the PI3K/Akt/mTOR pathway in the clinic

Several drugs targeting the PI3K/Akt/mTOR pathway have received regulatory approval in the treatment of a number of solid and hematological malignancies. Two of these agents, the mTOR inhibitor, everolimus, and α-specific PI3K inhibitor, alpelisib, are now established and licensed drugs for the treatment of advanced ER^+^ breast cancer when used in combination with ET [83–85]. See Table 1 for a summary of clinical trials combining ET with drugs targeting the PI3K/Akt/mTOR pathway.

**Table 1.** Published clinical trials combining ET and inhibitors of the PI3K/Akt/mTOR pathway

**Name of trial**	**Study design**	**Comparators**	**Primary endpoint**
mTOR inhibitors plus ET			
BOLERO-2 [86]	Phase III RCT 2 arms 724 patients	Everolimus/exemestane *vs.* placebo/exemestane	PFS 7.8 *vs.* 3.2 months HR 0.45; 95% CI: 0.38–0.54; *P* < 0.0001
BOLERO-6 [87]	Phase II RCT 3 arms 309 patients	Everolimus/exemestane *vs.* everolimus alone *vs.* capecitabine alone	PFS 8.4 *vs.* 6.8 *vs.* 9.6 months Everolimus/exemestane *vs.* everolimus: HR 0.74 (90% CI: 0.57–0.97) Everolimus/exemestane *vs.* capecitabine: HR 1.26 (90% CI: 0.96–1.66)
HORIZON [88]	Phase III RCT 2 arms 1,112 patients	Letrozole/temsirolimus *vs.* letrozole/placebo	PFS 8.9 *vs.* 9.0 months HR 0.90; 95% CI: 0.76–1.07; *P* = 0.25
TAMRAD [89]	Phase II open-label 2 arms 111 patients	Tamoxifen/everolimus *vs.* tamoxifen alone	6-month CBR 61% (95% CI: 47–74) *vs.* 42% (95% CI: 29–56); *P* = 0.045
PrE0102 [90]	Phase II RCT 2 arms 131 patients	Fulvestrant/everolimus *vs.* fulvestrant/placebo	PFS 10.3 *vs.* 5.1 months HR 0.61; 95% CI: 0.40–0.92; *P* = 0.02
NCT02049957 [91]	Phase Ib/II open-label 2 cohorts 118 patients	Everolimus-sensitive group sapanisertib/exemestane or fulvestrant *vs.* everolimus-resistant group sapanisertib/exemestane or fulvestrant	16-week CBR 45% (95% CI: 31.1–59.7) *vs.* 23% (95% CI: 11.8–38.6)
MANTA [92]	Phase II open-label 3 arms 333 patients	Fulvestrant/vistusertib (continuous or intermittent dosing) *vs.* fulvestrant/everolimus *vs.* fulvestrant alone	PFS 7.6 (daily vistusertib) and 8.0 (intermittent vistusertib) *vs.* 12.3 *vs.* 5.4 months Fulvestrant/daily vistusertib *vs.* fulvestrant: HR 0.88; 95% CI: 0.63–1.24; *P* = 0.46 Fulvestrant/intermittent vistusertib *vs.* fulvestrant: HR 0.79; 95% CI: 0.55–1.12; *P* = 0.16 Fulvestrant/daily vistusertib *vs.* fulvestrant/everolimus: HR 0.63; 95% CI: 0.45–0.90; *P* = 0.01 Fulvestrant/intermittent vistusertib *vs.* fulvestrant/everolimus: HR 0.71; 95% CI: 0.49–1.01; *P* = 0.06
TRINITI-1 [93]	Phase I/II open-label single-arm 95 patients	Ribociclib/everolimus/exemestane	CBR at week 24; 41.1% (95% CI: 31.1–51.6)
NCT02123823 [94]	Phase Ib/II open-label 2 arms 140 patients	Xentuzumab/everolimus/exemestane *vs.* everolimus/exemestane	PFS 7.3 *vs.* 5.6 months HR 0.97; 95% CI 0.57–1.65; *P* = 0.9057
PI3K inhibitors plus ET			
BELLE-2 [95]	Phase III RCT 2 arms 1,147 patients	Buparlisib/fulvestrant *vs.* placebo/fulvestrant	PFS total population 6.9 *vs.* 5.0 months HR 0.78; 95% CI: 0.67–0.89; *P* = 0.00021 PFS PI3K pathway-activated patients 6.8 *vs.* 4.0 months HR 0.76; 95% CI: 0.60–0.97; *P* = 0.014
BELLE-3 [96]	Phase III RCT 2 arms 432 patients	Buparlisib/fulvestrant *vs.* placebo/fulvestrant	PFS 3.9 *vs.* 1.8 months HR 0.67; 95% CI: 0.53–0.84; *P* = 0.00030
SOLAR-1 [97]	Phase III RCT 2 arms 572 patients	Alpelisib/fulvestrant *vs.* placebo/fulvestrant	PFS 11.0 *vs.* 5.7 months HR 0.65; 95% CI: 0.50–0.85; *P* < 0.001 in *PIK3CA*-mutant patients
NCT01870505 [98]	Phase I dose-escalation 2 arms 14 patients	Arm A: alpelisib/letrozole Arm B: alpelisib/exemestane	DLTs were maculopapular rash, hyperglycemia, and abdominal pain 8-week best response SD in 5 patients and PR in 1 patient
NCT01791478 [99]	Phase Ib Single-arm 26 patients	Letrozole/alpelisib	MTD of alpelisib plus letrozole at 300 mg/day CBR 35%; 95% CI: 17–56% (44% for *PIK3CA*-mutant *vs.* 20% for *PIK3CA* wild-type patients)
NCT01219699 [100]	Phase Ib open-label Single-arm 87 patients	Alpelisib/fulvestrant	MTD of alpelisib combined with fulvestrant 400 mg once daily, and the RP2D 300 mg PFS at the MTD 5.4 months (95% CI: 4.6–9.0)
FERGI [101]	Phase II RCT 2 arms, part 1 and 2 (only patients with *PIK3CA* mutations) 229 patients	Pictilisib/fulvestrant *vs.* placebo/fulvestrant	Part 1 PFS 6.6 *vs.* 5.1 months HR 0.74; 95% CI: 0.52–1.06; *P* = 0.096 Part 2 PFS 5.4 *vs.* 10.0 months HR 1.07; 95% CI: 0.53–2.18; *P* = 0.84
NCT01082068 [102]	Phase I/II open-label 2 arms 21 patients in phase I and 51 patients in phase II	Arm A: pilaralisib/letrozole Arm B: voxtalisib/letrozole	Arm A: ORR 4% (90% CI: 0.2–18.3) PFS 8 weeks (90% CI: 7.7–16.1) Arm B: no patient achieved ORR PFS 7.9 weeks (90% CI: 7.1–15.7)
BYlieve [103]	Phase II open-label 3 cohorts 127 patients	Alpelisib/fulvestrant	50.4% (95% CI: 41.2–59.6) alive without disease progression at 6 months
NCT02077933 [104]	Phase Ib open-label Breast cancer expansion cohort 11 patients	Alpelisib/exemestane with or without everolimus	Triplet escalation phase: MTD was alpelisib 200 mg, everolimus 2.5 mg, exemestane 25 mg Triplet cohort: ORR of 25.0% and DCR of 62.5% (90% CI: 28.9–88.9)
NCT02058381 [105]	Phase Ib open-label 2 arms 29 patients	Arm A: tamoxifen/goserelin/alpelisib Arm B: tamoxifen/goserelin/buparlisib	Arm A: treatment discontinuation 18.8%, PFS 25.2 months (95% CI: 2.7–36.3) Arm B: treatment discontinuation 53.8%, PFS 20.6 months (95% CI: 2.9 to not reached)
NEO-ORB [106]	Phase II RCT 2 arms 257 patients	Letrozole/alpelisib *vs.* letrozole/placebo	ORR 43% *vs.* 45% for *PIK3CA*-mutant patients and 63 *vs.* 61% for *PIK3CA* wild-type patients pCR 1.7% *vs.* 3% for *PIK3CA*-mutant patients and 2.8% *vs.* 1.7% for *PIK3CA* wild-type patients
NCT02734615 [107]	Phase I open-label, dose-escalation 3 arms 198 patients	Arm A: LSZ102 alone Arm B: LSZ102/ribociclib Arm C: LSZ102/alpelisib	Arm A: DLTs 5%, ORR 1.3% (95% CI: 0.0–7.0) Arm B: DLTs 3%, ORR 16.9% (95% CI: 9.3–27.1%) Arm C: DLTs 19%, ORR 7% (95% CI: 1.5–19.1)
SANDPIPER [108]	Phase III RCT 2 arms 516 patients	Taselisib/fulvestrant *vs.* placebo/fulvestrant	PFS 7.4 *vs.* 5.4 months HR 0.70; 95% CI: 0.56–0.89; *P* = 0.0037
NCT01296555 [109]	Phase II open-label single arm 60 patients	Taselisib/fulvestrant	CBR total population 29.5% (95% CI: 16.8–45.2) CBR *PIK3CA*-mutant 38.5% (95% CI: 13.9–68.4) ORR total population 22.7% (95% CI: 11.5–37.8) ORR *PIK3CA*-mutant 38.5% (95% CI: 13.9–68.4)
LORELEI [110]	Phase II RCT 2 arms 334 patients	Taselisib/letrozole *vs.* placebo/letrozole	ORR 38% for placebo *vs.* 50% for taselisib OR 1.55; 95% CI: 1.00–2.38; *P* = 0.049 pCR 2% for taselisib *vs.* 1% for placebo OR 3.07; 95% CI: 0.32–29.85; *P* = 0.37
PIPA [111]	Phase Ib expansion Single-arm 25 patients	Palbociclib/taselisib/fulvestrant	ORR 37.5% (95% CI: 18.8–59.4) CBR 58.3% (95% CI: 36.6–77.9) PFS 7.2 months (95% CI: 3.9–9.9)
NCT03006172 [112]	Phase I open-label, dose-escalation 2 arms 70 patients	Arm A: galone (GDC-0077)/letrozole Arm B: galone (GDC-0077)/palbociclib/letrozole	Arm A: no DLTs, confirmed PR 8%, CBR 35% Arm B: no DLTs, confirmed PR 36%, CBR 76%
Akt inhibitors plus ET			
FAKTION [113]	Phase II RCT 2 arms 140 patients	Capivasertib/fulvestrant *vs.* placebo/fulvestrant	PFS 10.3 *vs.* 4.8 months HR 0.58; 95% CI: 0.39–0.84; *P* = 0.0044
NCT01776008 [71]	Phase II open-label single-arm 16 patients	MK-2206/anastrozole plus goserelin for premenopausal patients	pCR rate 0% (90% CI: 0–17.1)
TAKTIC [114]	Phase Ib open-label 3 arms 25 patients	Arm A: ipatasertib/AI Arm B: ipatasertib/fulvestrant Arm C: ipatasertib/fulvestrant/palbociclib	Arm C (12 patients): no DLTs/discontinuations PR 2/12 patients SD 3/12 patients
Dual PI3K/mTOR inhibitors plus ET			
NCT02684032 [115]	Phase Ib dose-escalation/expansion 3 arms 35 patients	Arm A: gedatolisib/palbociclib/letrozole first-line Arm B: gedatolisib/palbociclib/fulvestrant second-line, CDKi 4/6 naive Arm C: gedatolisib/palbociclib/fulvestrant prior CDKi 4/6	Gedatolisib/palbociclib/letrozole DLTs 4/15 patients, SD/PR 53%/33% Gedatolisib/palbociclib/fulvestrant DLTs 4/20 patients, SD/PR 55%/20%

RCT: randomized controlled trial; PFS: progression-free survival; HR: hazard ratio; CBR: clinical benefit rate; DLT: dose-limiting toxicity; SD: stable disease; PR: partial response; MTD: maximum tolerated dose; ORR: objective response rate; pCR: pathologic complete response; CDKi: CDK inhibitor; DCR: disease control rate; RP2D: recommended phase 2 dose

### mTOR inhibitors

Since rapamycin (sirolimus) and the first generation of mTOR inhibitors were used in clinical practice, a wide range of agents have been developed, demonstrating more potent specificity [116]. Three generations of mTOR inhibitors have been studied for their effectiveness in different types of solid cancers. Rapamycin and its analogs (the rapalogs), temsirolimus, everolimus, and ridaforolimus, are allosteric inhibitors of mTOR, targeting the activity of the mTORC1 complex and inhibiting phosphorylation of downstream substrates [117]. Selective small-molecule mTORC2 inhibitors have been difficult to develop due to the intricate protein-protein interactions of the mTORC2 complex. Rapalogs lack sufficient mTORC2 inhibition and are known to stimulate the IGF-1 and Akt pathways through a feedback mechanism, ultimately leading to treatment resistance [118]. ATP-competitive mTOR inhibitors such as vistusertib and sapanisertib, and rapalink-1, are second and third-generation agents respectively, designed to overcome these issues by showing a higher affinity for both mTORC1 and 2 complexes [117, 119, 120]. The mTOR inhibitors temsirolimus and everolimus were the first to enter clinical practice in the treatment of advanced renal cell cancer, with indications later expanding to include ER^+^ HER2-negative (HER2^−^) breast cancer, pancreatic neuroendocrine tumors, and astrocytomas [121–124].

The combination of mTOR inhibition with ET has been extensively studied in the metastatic breast cancer setting. Following the positive results of the BOLERO-2 trial, everolimus was the first mTOR inhibitor to be approved in the treatment of breast cancer, when used in combination with exemestane [86]. This multicentre, phase III trial randomized 724 patients to receive either exemestane plus everolimus or exemestane plus placebo, with visceral disease and ET sensitivity as stratification factors. Clinical benefit was demonstrated for the combination, with a more than twofold increase in PFS compared to placebo, across all predefined subgroups although statistical significance was not achieved for its secondary endpoint, overall survival [125, 126]. BOLERO-6, a three-arm phase II study, compared everolimus plus exemestane *vs.* either everolimus or capecitabine alone. The study was powered to provide estimates of treatment effect, but no formal statistical analysis was preplanned. After a median follow-up of 37.6 months, PFS for the combination arm was estimated at 8.4 months *vs.* 6.8 months for exemestane monotherapy. Capecitabine outperformed everolimus plus exemestane, although the authors noted that this might be due to imbalances in the baseline characteristics [87]. Further supportive evidence of the combination of everolimus with ET was provided by the single-arm, phase II BOLERO-4 trial in which 202 patients were treated with letrozole plus everolimus in the first-line setting, and a median PFS of 22 months was observed [127]. The combination of everolimus plus exemestane in both the BOLERO-2 and BOLERO-4 trials demonstrated an acceptable safety profile. The main reported grade 3 and 4 adverse events (AE) were stomatitis, anemia, and fatigue, while hyperglycemia and pneumonitis were less frequent [86, 87].

Everolimus was further investigated in combination with tamoxifen, in the TAMRAD study, a phase II trial of metastatic, breast cancer resistant to AI therapy [89]. At 6 months, the CBR was shown to be significantly higher, compared to those treated with tamoxifen alone. The risk of progression and risk of death was also significantly reduced for patients receiving the combination, demonstrating a 4-month improvement in time to progression compared to monotherapy. In a subsequent predefined exploratory subgroup analysis, the response was found to be associated with acquired rather than primary hormone resistance, while further translational analysis of 55 tumor samples indicated mTORC1 activation as a potential predictive biomarker for treatment efficacy [89, 128]. The efficacy of another combination was assessed in the metastatic setting of AI-resistant ER^+^ breast cancer, comparing fulvestrant plus everolimus *vs.* fulvestrant plus placebo. PrE0102, a phase II randomized trial, reported a doubling of the PFS from 5.1 months to 10.3 months in favor of the combination. The objective response and CBRs showed a similar trend, whilst relatively few grade 3 and 4 AEs were observed [90].

In comparison, results from a phase III study of temsirolimus were disappointing. HORIZON compared first-line letrozole plus temsirolimus *vs.* letrozole plus placebo, for postmenopausal patients with advanced ER^+^ breast cancer [88]. The trial enrolled 1,112 patients, 40% of whom had received prior adjuvant ET. The primary endpoint, PFS, was not reached at the time of the second predefined interim analysis, and the study was consequently discontinued. Nevertheless, an interaction between age and treatment response was observed and further investigated in an exploratory analysis using subpopulation treatment effect pattern plot (STEPP) methodology, showing consistently improved outcomes in women aged ≤ 65 years (*P* = 0.003 for interaction). The authors concluded that the lack of PFS benefit could have been attributed to the difference in AI exposure between the populations recruited in HORIZON and BOLERO-2 trials, as well as the suspected alterations developed in endocrine-resistant tumors. In addition, the different drug formulations and dosing schedules between everolimus and temsirolimus, might have contributed to the contrasting findings [129].

Sapanisertib is a potent ATP-competitive inhibitor of mTORC1 and 2 and has been investigated in a non-randomized phase Ib/II study in which a total of 118 patients with metastatic, ER^+^ breast cancer that had previously progressed on everolimus with either exemestane or fulvestrant were recruited to receive sapanisertib in combination with exemestane or fulvestrant [91]. CBR was reported at 48% compared to 23% in the exemestane-sensitive and exemestane-resistant cohorts, respectively, with an overall response rate of 8% *vs.* 2%, respectively. Only a few patients exhibited dose-limiting toxicities, with nausea, diarrhea, fatigue, and hyperglycemia the most common AEs. Vistusertib, another dual mTORC1/2 inhibitor, has also been investigated in combination with fulvestrant in phase II randomized trial that recruited 333 post-menopausal women that had progressed on an AI [92]. The MANTA trial failed to meet its primary objective of PFS against both fulvestrant/everolimus combination and fulvestrant monotherapy.

### PI3K inhibitors

Buparlisib is an oral, potent, pan-PI3K inhibitor that has been extensively studied in both solid cancers and haematologic malignancies [84]. Its activity in ER^+^ breast cancer was investigated in two large, randomized, double-blind, multicentre phase III trials, BELLE-2 and BELLE-3. In the BELLE-2 trial, 1,147 postmenopausal women with advanced, AI-resistant breast cancer were randomized to receive daily doses of either buparlisib or placebo with monthly intramuscular fulvestrant, in 28-day cycles. The study met its primary endpoint, with a modest PFS benefit reported at 6.9 months for buparlisib *vs.* 5 months for the control arm. However, the authors concluded that in view of the increased toxicities observed in the buparlisib group, including liver toxicity, rash, and hyperglycemia, no further study of this combination should be pursued [95]. Results of the BELLE-3 trial, published soon after, led to a similar conclusion. In this placebo-controlled trial, the combination of buparlisib plus fulvestrant was investigated in patients who had progressed on or after ET and mTOR treatment. Again, a two-month PFS benefit was demonstrated in favor of buparlisib (3.9 months *vs.* 1.8 months). However, the toxicity profile of buparlisib proved unacceptable and two treatment-related deaths were attributed to the combination [96].

Pictilisib, a pan-PI3K inhibitor with higher affinity for the α and δ isoforms, was studied in combination with fulvestrant in the FERGI trial a randomized, placebo-controlled phase II trial that recruited patients with metastatic breast cancer resistant to ET. Patients were stratified according to *PIK3CA* mutation status and previous exposure to AI, leading to primary or secondary resistance. However, no difference in PFS was achieved in any of the subgroups treated with the combination, and tolerability was challenging [101]. Pilaralisib, another pan-PI3K inhibitor, as well as the dual PI3K/mTOR inhibitor voxtalisib were each tested in combination with letrozole in phase I/II trial of AI-refractory breast cancer. However clinical activity was disappointing and among the 25 and 26 patients enrolled in phase II, in the pilaralisib and voxtalisib arms respectively, only one treated with pilaralisib achieved a PR with a median PFS of 8 weeks [102].

The need for improved efficacy and tolerability has led to a shift in interest toward isoform-specific PI3K-inhibitors. Alpelisib selectively inhibits the PI3Kα isoform and its effectiveness against tumors harboring *PIK3CA* mutations was initially established in several early phase studies, testing the combination of alpelisib with ET in patients with ER^+^ breast cancer [98, 100]. The publication of results from the pivotal phase III SOLAR-1 trial eventually led to the approval of alpelisib in combination with fulvestrant for patients with *PIK3CA*-mutant, ER^+^ advanced breast cancer, and prior exposure to ET [85]. In this pivotal study, the cohort of 341 patients with *PIK3CA* mutations showed a significantly improved PFS of 11 months in the fulvestrant/alpelisib group *vs.* 5.7 months in the fulvestrant/placebo group meeting its primary endpoint. The most common grade 3/4 toxicities involved hyperglycemia, rash, and diarrhea, which attributed to an increased treatment discontinuation rate in patients receiving the combination. Final overall survival results were recently published, showing a difference of 7.9 months in favor of alpelisib, but failing to reach statistical significance. Nonetheless, a stronger treatment effect with borderline significance was observed in patients with lung and/or liver disease for alpelisib [97].

Although prior treatment with CDK4/6 inhibitor (CDK4/6i) was one of the stratification factors in SOLAR-1, only 20 patients were identified in this subgroup, with the HR for progression or death not reaching statistical significance (HR 0.48; 95% CI: 0.17–1.36). However, the BYlieve study, a phase II cohort study of 127 patients with *PIK3CA* mutations pretreated with a CDK4/6i, was recently published showing 50.4% of patients were without progression or death at 6 months. Although there was no control arm, these data provide some support for the use of alpelisib/fulvestrant as a therapeutic option in patients with *PIK3CA*-mutated disease following progression on first-line CDK4/6i [103]. In addition, alpelisib has been investigated as part of a triplet regimen in combination with everolimus and exemestane in a phase Ib study of postmenopausal women with ER^+^ breast cancer, with an acceptable toxicity profile [130]. Another phase Ib study tested the combination of tamoxifen and goserelin acetate with either alpelisib or buparlisib in premenopausal women with advanced breast cancer [131]. Poor tolerability was observed with buparlisib and here most patients were discontinued due to AEs. A randomized phase II study of alpelisib in combination with letrozole *vs.* letrozole alone showed no improvement in response in the neoadjuvant setting [106, 132].

Lastly, the effectiveness of taselisib, a potent PI3Kα-inhibitor, was assessed in a single-arm, phase II study, in combination with fulvestrant in patients with advanced ER^+^ breast cancer with encouraging activity [109]. Further to this exploratory data, the SANDPIPER study recruited 516 patients with endocrine-resistant, *PIK3CA*-mutant breast cancer to fulvestrant/taselisib *vs.* fulvestrant/placebo and met its primary endpoints of a statistically significant improvement in PFS (7.4 months *vs.* 5.4 months in favor of taselisib). However, given the high rate of serious AEs in the taselisib arm and the modest clinical benefit, taselisib has not become an established therapeutic option [108]. The combination of taselisib/letrozole *vs.* placebo/letrozole has also been explored in the neoadjuvant setting, in the randomized phase II LORELEI trial with a higher ORR observed for the taselisib combination [110].

### Akt inhibitors

In the clinic the most extensively studied Akt inhibitors are capivasertib and ipatasertib, both ATP-selective pan-Akt inhibitors with activity against Akt 1, 2, and 3. The FAKTION randomized phase II study examined the addition of capivasertib to fulvestrant *vs.* placebo in 140 women with ER^+^ metastatic breast cancer resistant to an AI. PFS was significantly prolonged in the combination arm (10.3 months *vs.* 4.8 months in favor of capivasertib) and frequent grade 3/4 AEs in the capivasertib arm were hypertension, diarrhea, rash, and infection [113]. An ongoing phase III trial, CAPItello-291, designed to further evaluate this combination, is currently recruiting [133]. Ipatasertib is currently under investigation in combination with fulvestrant in the ongoing, phase III FINER trial, in patients who progressed after first-line treatment with a CDK4/6i and an AI (NCT04650581) [134]. The allosteric pan-Akt inhibitor, MK-2206, was assessed in combination with anastrozole in the neoadjuvant setting in a phase single-arm II study of patients with *PIK3CA*-mutant disease but clinical activity was not encouraging and toxicity significant [71].

### Agents targeting IGF-1 axis

Several clinical trials assessing agents that target IGF-1 signaling in combination with ET are ongoing in the setting of both endocrine-sensitive and resistant diseases [77, 135].

Xentuzumab is a humanized, IGF-1 and IGF-2 neutralizing antibody that has been tested in combination with everolimus and exemestane in a phase Ib/II trial of advanced, ER^+^ breast cancer. The triplet regimen was administered to 24 post-menopausal women with the endocrine-resistant disease in phase Ib of the study and was well-tolerated, with disease control observed in 57% of patients, and PRs reported in 19% [136]. In a phase II study, patients were randomized to receive either the triplet regimen or the exemestane/everolimus doublet but no PFS benefit was observed in the overall population, although in a pre-specified subgroup analysis there was a suggestion of benefit in patients without the visceral disease [94]. XENERA-1, a phase II trial also assessing this combination, is ongoing in patients with the non-visceral disease. The addition of xentuzumab to abemaciclib, a CDK4/6i, in combination with fulvestrant, has also been investigated in a phase Ib study of women with the endocrine-resistant disease and no prior treatment with CDK4/6i or chemotherapy. Early data suggest encouraging clinical activity and tolerability [137]. A phase II study of another IGF-1/2 neutralizing monoclonal antibody, dusigitumab, in combination with an AI in ER^+^ breast cancer is yet to report [80].

A different approach involves the employment of monoclonal antibodies directed toward the IGF-1R. Three agents, ganitumab, cixutumumab, and dalotuzumab have been tested in phase II trials in combination with mTOR inhibitors and ET, with no supportive evidence of their treatment benefit to date [138–141]. In addition to their limited efficacy, the AE profile, in particular hyperglycemia and hyperinsulinemia, have complicated clinical development [77].

In summary, the mTOR inhibitor, everolimus, and PI3Kα inhibitor, alpelisib, are now licensed therapies that have been shown to improve treatment outcomes for metastatic breast cancer when used in combination with hormonal therapy. Other avenues of investigation are ongoing, in particular combining either Akt inhibitors or agents targeting IGF-1 signaling with hormonal therapy.

## Molecular markers and resistance pathways to PI3K/Akt/mTOR inhibition

The clinical development of therapeutics that target the PI3K/Akt/mTOR pathway in ER^+^ breast cancer has met with significant challenges. PI3K/Akt/mTOR activation is not ubiquitous in ER^+^ breast cancer and can happen at several different nodes in the pathway as described above. PI3K/Akt/mTOR pathway blockade is associated with a variety of significant toxicities, including hyperglycemia, which, as described below, may be a mechanism of resistance. Additionally, multiple other resistance mechanisms exist, both intrinsic to the PI3K/Akt/mTOR pathway and in related pathways. Identification of suitable predictive biomarkers is therefore paramount for providing a personalized approach to treatment and to amplifying the chances of demonstrating efficacy in late-phase clinical trials.

### Biomarkers that predict response to inhibition of the PI3K/Akt/mTOR inhibition

The utility of predictive biomarkers is, in part, dependent on the target of interest within the PI3K/Akt/mTOR pathway. mTOR inhibitors were the first treatment class to be licensed and, as described above, everolimus has shown clinical benefit when given in combination with exemestane for metastatic ER^+^ breast cancer [142]. However, many patients do not benefit, and significant toxicities are a major factor in discontinuing therapy. Despite the importance of patient selection, there is still no established predictive biomarker in the clinic, though ER^+^/HER2 positive (HER2^+^) and ER^+^ basal-like subtypes appeared to fare worse in retrospective analyses [126, 143–145]. A retrospective study of patients receiving everolimus/exemestane suggested *AKT1*^E17K^ mutations may predict longer PFS, although this needs prospective investigation [146].

Preclinical work identified mutations within *PIK3CA*, the gene encoding the PI3Kα isoform p110α catalytic subunit, as an early potential predictive biomarker for PI3K inhibitors [65, 147]. Multiple PI3K inhibitors have been developed for solid tumors, though as previously described, pan-PI3K inhibition (PI3K isoforms α, β, γ, and δ) in ER^+^ breast cancer has yielded negative trial results [95, 96, 101, 148, 149]. There is also some evidence for the predictive value of *PIK3CA* mutations for the pan-class I PI3K inhibitor, buparlisib, in metastatic ER^+^ breast cancer [95, 96]. Assaying for *PIK3CA* as a predictive biomarker for this therapeutic class has been inconsistent, both in approach and results. The pan-PI3K inhibitor trials used varied biomarkers including assessing *PIK3CA* hotspot mutations within exons 1, 4, 7, 9, and 20, specific point mutations within these exons such as *PIK3CA*^E545K^, or composite “PI3K pathway activation” that included either *PIK3CA* mutation or PTEN loss of function. Additionally, the *PIK3CA* mutational analysis included both tumor biopsy samples and circulating tumor DNA (ctDNA), further complicating matters.

The development of isoform-specific inhibitors followed the early failures of pan-PI3K inhibitors in ER^+^ breast cancer, with improved efficacy and toxicity profiles [85, 108]. As discussed above, the SOLAR-1 trial showed that *PIK3CA* mutations, detected either using ctDNA or in tumor tissue, were predictive of improved PFS for alpelisib plus fulvestrant [85].

Results for taselisib plus fulvestrant in the SANDPIPER-3 trial in metastatic ER^+^ breast cancer were less clear. Although *PIK3CA* mutations were predictive for a statistically significant improvement in PFS, the HR in the *PIK3CA* wild-type group was essentially the same as the mutant group, albeit lacking statistical significance [108]. In both trials, the predictive value of activating *PIK3CA* mutations was independent of both site and type of mutation. Despite their predictive value in metastatic disease, *PIK3CA* mutations were not predictive in neoadjuvant trials of either alpelisib or taselisib [106, 110].

Potential predictive biomarkers, other than *PIK3CA*, have also been investigated for PI3K inhibitors. Preclinical evidence suggests that PTEN loss should sensitize cells to PI3K/Akt inhibition, although this has not been demonstrated unambiguously in the clinic for either pan-PI3K or PI3Kα inhibitors [65, 95, 149, 150]. Alterations in the p85 regulatory subunit of PI3K due to mutations can lead to constitutive hyperactive PI3K/Akt signaling and this genetic alteration is relatively uncommon in breast cancer (2.8% in the TCGA database) [151]. Luminal B subtype and progesterone receptor-negative disease both appeared to be predictive of Ki67 suppression for pictilisib in the neoadjuvant setting [150]. However, this was only in a small, negative, phase II study, and not seen in trials of other PI3K inhibitors.

Preclinical evidence suggests mutations in *PIK3CA*, *PIK3R1*, *Akt*, or mutation/loss of PTEN could predict treatment response for Akt inhibitors [152–154]. However, although predictive *in vitro* and *in vivo*, *PIK3CA* and *PTEN* alterations have not been predictive of treatment response in clinical trials of these agents [71, 113, 155–157]. In an early phase single cohort study, fulvestrant combined with the Akt inhibitor, capivasertib, has shown encouraging clinical activity in heavily pretreated patients with ER^+^ breast cancer and *AKT1*^E17K^ mutations [158]. There are ongoing phase III trials investigating biomarker-driven response and survival for capivasertib and ipatasertib, with stratification according to PI3K/PTEN/Akt alterations, which will hopefully provide clear evidence for a biomarker-driven approach [133, 159].

### Mechanisms of resistance to PI3K/Akt/mTOR inhibition

Understanding primary or secondary resistance to targeted therapies can inform the patient selection and novel combinations. Resistance mechanisms may be intrinsic to the pathway or arise through extrinsic, related signaling. For example, mTOR’s auto-regulatory feedback loop attenuates its own response, with inhibition of mTORC1 leading to increased activity of PI3K [160]. For PI3K inhibitors, constitutive activity of any of the regulatory or downstream effector proteins has the potential to mediate resistance. PTEN loss has been proposed as a key mechanism of resistance to inhibitors directed at PI3K pathway but not pan-PI3K inhibitors [149, 161]. PTEN loss leads to cells becoming dependent on PI3Kβ, which may explain this differential response to therapy [162]. Activating Akt mutations may also define resistance to PI3K inhibitors, with some early evidence of re-sensitization to PI3K inhibitors with the addition of an Akt inhibitor in a translational study [163].

The EGFR/Ras/Raf/MAPK kinase (MEK)/ERK and PI3K/Akt/mTOR signaling cascades co-regulate many downstream effectors in parallel. The presence of Kirsten RAS 2 viral oncogene homolog (*KRAS*) mutations associates with *PIK3CA* mutations in cancer. *KRAS* mutations appear to confer resistance to both alpelisib and ipatasertib in early phase trials [164, 165]. While combination inhibition with alpelisib, cetuximab, encorafenib, and binimetinib has been trialed in B-Raf proto-oncogene, serine/threonine kinase (*BRAF*)-mutant colorectal cancer, evidence for ER^+^ breast cancer is lacking [166]. For alpelisib, tumor protein p53 (TP53) mutation and fibroblast growth factor receptors (FGFR) 1 and 2 amplification have also been proposed as markers of resistance [99].

### Insulin and resistance to PI3K inhibitors

**Figure 2.**
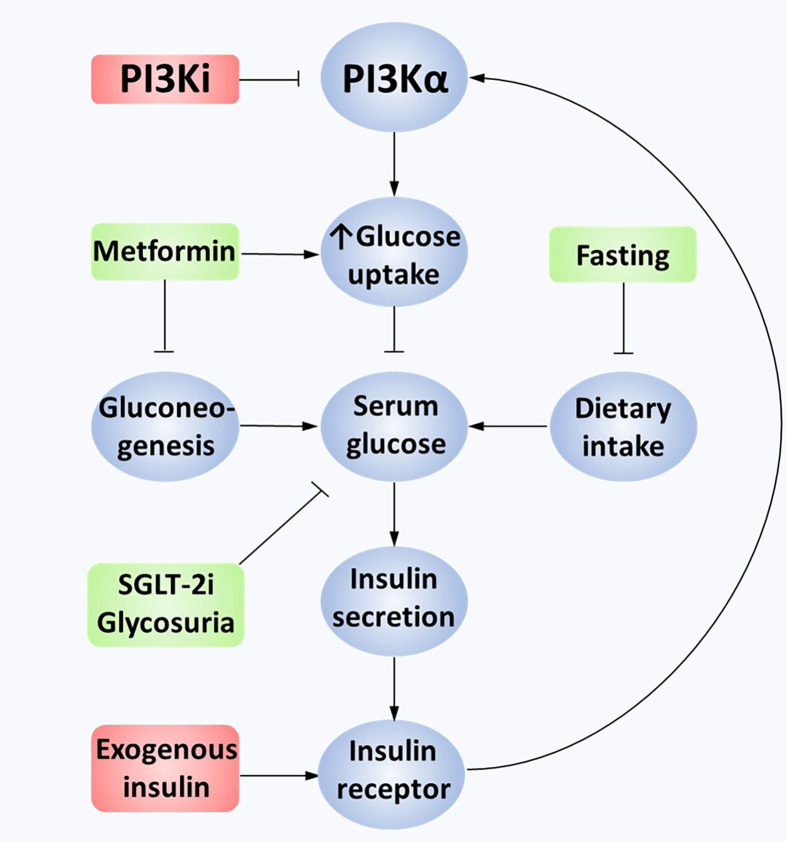
Mechanisms of hyperglycemia-mediated PI3K inhibitor resistance. Constitutively active PI3Kα leads to the transport of GLUT4 vesicles to the cell membrane, causing glucose uptake into cancer cells. PI3K inhibition limits downstream pathway activation in tumor and non-tumor cells. GLUT4 vesicles are no longer transferred to the cell membrane as an on-target negative consequence, leading to extracellular hyperglycemia. This stimulates excess insulin secretion in pancreatic beta cells, binding to IRs on cancer cells. Overactivation of the IR overcomes the PI3Ki effect, partially reactivating the PI3K/Akt/mTOR pathway. This effect can also be mediated by exogenous insulin. Minimizing hyperglycemia through fasting, metformin or SGLT-2 inhibition reduces insulin secretion, restoring the effectiveness of PI3K inhibition. GLUT4: glucose transporter type 4; PI3Ki: PI3K inhibition

Hyperglycaemia is a common on-target toxicity of PI3K inhibitors. PI3K is a necessary downstream effector of the IR and hence inhibition leads to insulin resistance [167]. The resultant increase in circulating glucose further increases insulin secretion. A detailed preclinical study showed that higher levels of circulating insulin as a result of PI3K inhibition may act as a resistance mechanism [168]. The excess insulin secreted in response to hyperglycemia increases signal transduction from the IR to PI3K, abrogating the PI3K inhibitor’s antagonism and restoring pro-oncogenic downstream signaling. Using *in vivo* patient-derived xenograft mouse models, this study showed that metformin, sodium-glucose cotransporter-2 (SGLT-2) inhibitors, and a ketogenic diet were all capable of restoring inhibition of PI3K/Akt/mTOR signaling. Exogenous insulin nullified the benefit of a ketogenic diet in these mice, suggesting insulin should be avoided in managing this toxicity. To date, in the clinic, standard practice for managing hyperglycemia has been to use metformin [85, 108, 169]. However, it was notable in this study that SGLT-2 inhibition led to a greater restoration of PI3K-inhibitor sensitivity than metformin [168]. A case report of the use of SGLT-2 inhibitors with alpelisib suggests this is well tolerated and can prevent the discontinuation of alpelisib [170]. Of note SGLT-2 inhibitors are associated with urinary tract infections and very rare, life-threatening toxicities including euglycaemic diabetic ketoacidosis, hence safety studies in combination with PI3K inhibitors would help inform practice [171, 172]. See Figure 2 for an overview of hyperglycemia-mediated PI3K inhibitor resistance.

Calorie restriction and fasting have long been recognized as limiting tumor growth in animal studies [173] and it has been proposed that this is due to reduced insulin-mediated PI3K/Akt/mTOR signaling [174, 175]. Different methods of calorie restriction have been assessed in both animal and human studies [176]. One approach is the fasting-mimicking diet (FMD) which is a low-calorie plant-based diet, high in fat, and low in protein and carbohydrate with micronutrient supplementation [177]. FMD lasts for 4 days, followed by a recovery diet for 26 days. Preclinical work in ER^+^ breast cancer showed that FMD could induce cancer cell regression, both *in vitro* and *in vivo* [178]. Subsequent early phase studies have demonstrated that FMD is tolerable and safe in patients with a variety of cancers, including breast cancer [177]. FMD alone would not be sufficient for restoring endocrine sensitivity in all breast cancers, as tumors with constitutive activation of PI3K/Akt/mTOR signaling are likely to be resistant to fasting approaches [179]. However, fasting approaches in combination with FMD with ET warrant further study in clinical trials.

In summary, multiple mechanisms of resistance and several putative biomarkers have been identified for PI3K and mTOR inhibitors in breast cancer, mostly focusing on specific genetic alterations. However, new data suggests that the feedback hyperglycemia from targeting this pathway may also play a key role in resistance to treatment and hence therapeutic opportunity.

## Future perspectives

As new drugs for the treatment of ER^+^ breast cancer continue to emerge, there may be further opportunities for efficacious novel combinations with mTOR and PI3K inhibitors in appropriately selected populations. To date, published data from clinical trials have used fulvestrant, AI, or tamoxifen as the ET backbone to mTOR, Akt, or PI3K inhibition. Fulvestrant is the only SERD that is licensed for clinical practice but novel, oral SERDs are now in clinical development with improved pharmacokinetics and bioavailability [180]. Four agents have already entered phase III testing; amcenestrant, camizestrant, and giredestrant are examined in combination with palbociclib in the AMEERA-5, SERENA-4, and persevERA breast cancer trials respectively, while elacestrant monotherapy is tested *vs.* fulvestrant or AI in patients who progressed on CDK4/6i (EMERALD trial) [181]. Early phase studies are assessing combinations of novel SERDs with inhibitors of the PI3K/Akt/mTOR pathway. AMEERA-1, a phase Ia/b trial of amcenestrant in combination with alpelisib and everolimus, is actively recruiting. Similarly, the ongoing SERENA-1 trial is testing camizestrant alongside everolimus or capivasertib (NCT03616587). The only currently available evidence comes from another phase I study, investigating the oral SERD LSZ102. In one of the experimental arms, 43 patients with endocrine-resistant breast cancer were treated with LSZ102 plus alpelisib. Clinical activity was encouraging, with ORR and CBR measured at 7.0% (95% CI: 1.5–19.1) and 20.9% (95% CI: 10.0–36.0) respectively, with an estimated modest median PFS of 3.5 months, irrespective of PIK3CA mutation status [107].

Co-targeting of the ER and CDK4/6 has become the standard of care for patients with advanced, ER^+^ breast cancer, albeit acquired resistance to CDK4/6i still almost inevitably develops [182]. Preclinical data have suggested potential synergy in targeting PI3K/Akt/mTOR and CDK4/6 signaling [183]. Preliminary results were published from a phase Ib trial assessing the addition of gedatolisib, a dual PI3K-mTOR inhibitor, to palbociclib plus either letrozole or fulvestrant. Early data suggests promising clinical activity for this combination alongside a manageable toxicity profile, with nausea, neutropenia, and stomatitis as the most frequent AEs [115]. Similarly, recent results of an early phase trial, investigating the combination of inavolisib, a PI3Kα inhibitor, and letrozole with or without palbociclib in patients with *PIK3CA*-mutant disease, suggested augmented anti-tumor activity with the triplet regimen. Confirmed PR and CBR were measured at 36% and 76%, respectively, for the inavolisib combination, while hyperglycemia, stomatitis, gastrointestinal, and hematological toxicities were among the most common AEs. Further insight into the potential of this triplet combination is expected to be provided by a phase III trial that is currently recruiting (NCT04191499) [112].

Taselisib has also been tested as a triplet therapy along palbociclib and fulvestrant in *PIK3CA*-mutant, ER^+^ breast cancer in the single-arm PIPA trial. The authors concluded that a response rate of 37.5% was promising for superiority to the palbociclib/fulvestrant doublet and warrants further investigation [111]. The Akt inhibitor ipatasertib combined with palbociclib/fulvestrant is under investigation in the phase Ib/III trial, IPATunity150 (NCT04060862) [158]. This placebo-controlled study in the first-line setting of endocrine-resistant breast cancer will add to the existing evidence of the phase Ib TAKTIC trial, whose interim results of 12 patients treated with this triplet combination displayed manageable tolerability and some promise of clinical benefit [114]. Finally, the phase I/II TRINITI-1 trial has just reported results of the combination everolimus/exemestane/ribociclib in 104 patients who progressed on CDK4/6i [93]. The favorable toxicity profile and the observed clinical benefit, with CBR at 41.1%, warrant further study.

In the clinic, prospective future directions for targeting PI3K/Akt/mTOR in ER^+^ breast cancer also include further combinations with IGF-targeted agents, antagonists of the androgen receptor (AR), FGFR inhibitors, and checkpoint immunotherapy. As outlined above, data from clinical studies of xentuzumab and other anti-IGF-1R antibodies have, however, been disappointing to date. The role of AR expression in breast cancer and the efficacy of its inhibition has been better understood and established in the ER-negative disease [184]. Preclinical breast cancer models have associated increased AR levels with the presence of *PIK3CA* mutations and have suggested prognostic value in breast cancer, irrespective of the hormonal status [185, 186]. Further evidence of increased sensitivity of AR-positive (AR^+^) breast cancer to PI3K pathway inhibition and its potential as a predictive biomarker has provided a rationale for the combined use of anti-androgens with drugs targeting PI3K/Akt/mTOR [187, 188]. An early-phase trial exploring the safety and efficacy of alpelisib in combination with enzalutamide is ongoing for patients with metastatic breast cancer that is AR^+^ with PTEN loss (NCT03207529) [189].

Another approach takes advantage of the interplay between the fibroblast growth factor (FGF)/FGFR and PI3K/Akt/mTOR pathways, as FGFR overexpression leads to activation of PI3K/Akt signaling [190]. Aberrant expression of FGFR has been identified as a mediator of endocrine resistance and hence targeting both pathways, especially in the presence of concurrent genetic alterations, is an attractive strategy to augment the efficacy of the PI3K/Akt/mTOR inhibitors [191, 192]. In light of this evidence, a phase I trial studied the combination of alpelisib plus infigratinib in patients with *PIK3CA*-mutant solid cancers. Unfortunately, at this point results were disappointing without indication of significant clinical activity [193].

## Conclusions

In summary, targeting the PI3K/Akt/mTOR pathway to subvert resistance to ET in breast cancer is now proven to be of clinical benefit with everolimus and alpelisib already used routinely in clinical practice. A new generation of therapeutics awaits full evaluation in this setting, including drugs targeting Akt and IGF-1 signaling. However, challenges remain, in particular, the need for the development of improved biomarkers for patient selection and clinical evaluation of strategies to abrogate mechanisms resistance, such as feedback hyperglycemia.

## Abbreviations

AE: adverse events
AI: aromatase inhibitors
AR: androgen receptor
CBR: clinical benefit rate
CDK4/6: cyclin-dependent kinases 4 and 6
CDK4/6i: cyclin-dependent kinases 4 and 6 inhibitor
CI: confidence interval
DLT: dose-limiting toxicity
ER: estrogen receptor
ER^+^: estrogen receptor-positive
ET: endocrine therapy
FGFR: fibroblast growth factor receptors
FMD: fasting-mimicking diet
HER3: human epidermal growth factor receptor 3
HR: hazard ratio
IGF: insulin growth factor
IGF-1R: insulin growth factor-1 receptor
IGFR: insulin growth factor receptor
IR: insulin receptor
IRS-1: insulin receptor substrate-1
MAPK: mitogen-activated protein kinase
mTORC1: mechanistic target of rapamycin complex 1
OR: odds ratio
ORR: objective response rate
PDK1: 3-phosphoinositide-dependent protein kinase-1
PFS: progression-free survival
PIP_2_: phosphatidylinositol 4,5-bisphosphate
PIP_3_: phosphatidylinositol-3,4,5-trisphosphate
PR: partial response
PTEN: phosphatase and tensin homolog
RAS: rat sarcoma
S473: serine 473
SERD: selective estrogen receptor degraders
SERMs: selective estrogen receptor modulators
SGLT-2: sodium-glucose cotransporter-2
T308: threonine 308

## Declarations

### Copyright

© The Author(s) 2022.
